# Growth, respiratory activity and chlorpyrifos biodegradation in cultures of *Azotobacter vinelandii* ATCC 12837

**DOI:** 10.1186/s13568-021-01339-w

**Published:** 2021-12-27

**Authors:** Victoria Conde-Avila, Carlos Peña, Beatriz Pérez-Armendáriz, Octavio Loera, Carmen Martínez Valenzuela, José Belisario Leyva Morales, Pedro de Jesús Bastidas Bastidas, Holjes Salgado-Lugo, Luis Daniel Ortega Martínez

**Affiliations:** 1 Facultad de Biotecnología, Universidad Popular Autónoma del Estado de Puebla, México, 13 Poniente No. 1927 Col. Barrio de Santiago, 72410 Puebla, Pue México; 2 Departamento de Ingeniería Celular y Biocatálisis, Instituto de Biotecnología, Universidad Nacional Autónoma de México, Apdo. Post. 510-3, Cuernavaca, 62250 Morelos México; 3 Universidad Autónoma Metropolitana-Unidad Iztapalapa, Av. San Rafael Atlixco 186, 09340 Mexico City, CDMX México; 4 Doctorado en Sustentabilidad, Universidad Autónoma de Occidente, Unidad Guasave. Avenida Universidad S/N, Fraccionamiento Villa Universidad, 81048 Guasave, Sinaloa México; 5 Centro de Investigación en Alimentación y Desarrollo, A.C. (Unidad Culiacán), Carretera a El dorado km. 5.5, Campo el Diez, 80129 Culiacán, Sinaloa México

**Keywords:** Oxygen consumption rate, Pesticide degradation, Respiratory quotient, Rhizobacteria

## Abstract

This study aimed to evaluate the growth, respiratory activity, and biodegradation of chlorpyrifos in cultures of *Azotobacter vinelandii* ATCC 12837. A strategy based on the modification of culture media and aeration conditions was carried out to increase the cell concentration of *A. vinelandii*, in order to favor and determine its tolerance to chlorpyrifos and its degradation ability. The culture in shaken flasks, using sucrose as a carbon source, significantly improved the growth compared to media with mannitol. When the strain was cultivated under oxygen-limited (5.5, 11.25 mmol L^−1^ h^−1^) and no-oxygen-limited conditions (22 mmol L^−1^ h^−1^), the growth parameters were not affected. In cultures in a liquid medium with chlorpyrifos, the bacteria tolerated a high pesticide concentration (500 ppm) and the growth parameters were improved even under conditions with a reduced carbon source (sucrose 2 g L^−1^). The strain degraded 99.6% of chlorpyrifos at 60 h of cultivation, in co-metabolism with sucrose; notably, *A. vinelandii* ATCC 12837 reduced by 50% the initial pesticide concentration in only 6 h (DT_50_).

**Supplementary Information:**

The online version contains supplementary material available at 10.1186/s13568-021-01339-w.

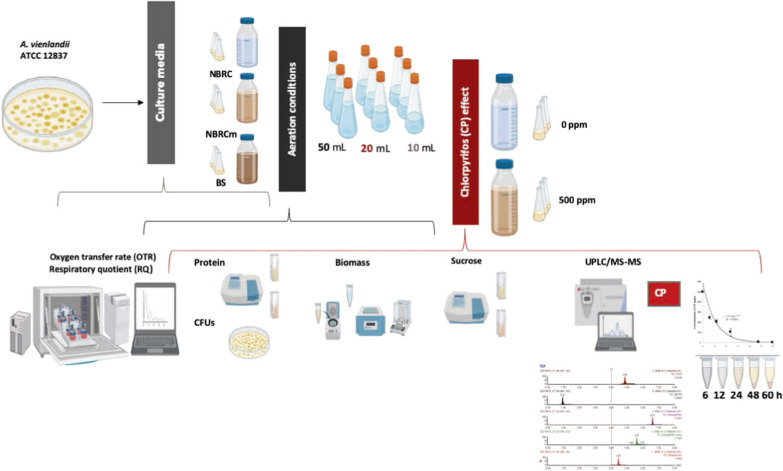

## Key Points

- *A. vinelandii* ATCC 12837 tolerates, grows, and degrades high concentrations of chlorpyrifos in vitro.
- Respirometric parameters of *A. vinelandii* ATCC 12837 were not adversely affected by chlorpyrifos.
- The use of a sucrose-enriched medium favored the biodegradation of chlorpyrifos by *A. vinelandii*.

## Introduction

One of the ecotoxicological problems caused by the intensive use of organophosphate pesticides (OP) is damage to non-target organisms. Pesticides can inhibit the growth of beneficial microorganisms, such as plant growth-promoting rhizobacteria (PGPR) (Walvekar et al. 2017), or reduce metabolic capacities related to their efficacy as inoculants (Sethi and Gupta 2013; Abo-amer et al. 2014; Muttawar and Wadhai 2014).

The evaluation of the effects of the most widely used OP worldwide on PGPR has gained interest because tolerant organisms could maintain their promoting activities, establish in contaminated sites, even used as potential decontaminating agents (Sumbul et al. 2020; Chitara et al. 2021). Tolerance and degradation to various pesticides by PGPR have been evaluated in the genera *Azospirillum* (Santos et al. 2020), *Bacillus* (Praveen Kumar et al. 2014), *Klebsiella* (Rani et al. 2019), *Pseudomonas* (Giri and Rai 2012), *Serriata* (Cycón et al. 2013)*, Ochrobactrum* (Abraham and Silambarasan 2016) and *Azotobacter* (Chennappa et al. 2018a, b), the latter being one of the most important for agricultural proposes.

*Azotobacter* spp. are efficient in asymbiotic N_2_ fixation, P solubilization (Sethi and Gupta 2013), production of phytohormones (Chobotarov et al. 2017), siderophores (Shahid et al. 2019), vitamins (Revillas et al. 2000), synthesis of antimicrobial compounds (Nagaraja et al. 2016), production of metabolites of industrial interest such as the alginate and polyhydroxybutyrate (PHB) (Gurikar et al. 2016), as well as in the synthesis of enzymes involved in degradation processes of toxic substances (Chennappa et al. 2019).

Some *Azotobacter* species degrade aromatic compounds such as insecticides, fungicides, and herbicides (Castillo et al. 2011; Chennappa et al. 2016). These bacteria have particularly shown tolerance to endosulfan, phorate, carbendazim, chlorpyrifos (CP), pendimethalin, among others (Castillo et al. 2011; Chennappa et al. 2014a; Gurikar et al. 2016; Rani and Kumar et al. 2017), without showing growth inhibition (Chennappa et al. 2016). Also, there are reports describing the degradation of lindane (Anupama and Paul 2009), phorate (Moneke et al. 2010), endosulfan (Castillo et al. 2011), pendimethalin (Chennappa et al. 2018a), glyphosate (Mousa et al. 2021), and CP by *Azotobacter* isolates (Chennappa et al. 2019).

In contrast, other authors have reported adverse effects for *Azotobacter* spp. (Askar and Khudhur 2013; Chennappa et al. 2013; Walvekar et al. 2017; Kumar et al. 2019); e.g. reduced growth rate in the presence of CP (Menon et al. 2004), glyphosate (Moneke et al. 2010) and endosulfan (Castillo et al. 2011), inhibition of diazotrophic activity (Menon et al. 2004; Chennappa et al. 2019), reduced respiration rate with glyphosate, pendimethalin and fomesafen (Chennappa et al. 2013, 2014b; Wu et al. 2014), cell damage and loss of viability after exposure to different concentrations of glyphosate and atrazine (Shahid et al. 2019).

The genus *Azotobacter* can exhibit varied behaviors depending on the species and strains, growth conditions, type of pesticide, and contaminant concentrations; therefore, it is useful to evaluate the effect of these factors on model organisms such as *Azotobacter vinelandii* (Noar and Bruno-Bárcena 2018); *A. vinelandii* is a strictly aerobic free-living bacterium with growth and metabolite production, both in vitro and in soil, closely related to physicochemical parameters (Lenart 2012; Plunkett et al. 2020), nutrient concentration and availability (essentially carbon and nitrogen sources) (Tejera et al. 2005; Then et al. 2016), microbial interactions (Bhosale et al. 2013), exposure to toxic substances (Chennappa et al. 2019) and oxygenation levels (Peña et al. 2007; Castillo et al. 2013), the latter being one of the critical parameters because of the high oxygen rate consumption of *Azotobacter* spp. On this regard, some aspects of the respiration in *A. vinelandii* have been evaluated widely concerning its growth and polymers synthesis (Lozano et al. 2011; Castillo et al. 2020). Culture factors such as the oxygen transfer rate (OTR) and respiratory quotient (RQ) are crucial in describing the physiological state under different growth conditions. They are related to parameters such as the specific growth rate and metabolite production (Gómez-Pazarín et al. 2015). Additionally, it can be useful for monitoring degradation processes (Kahraman and Altin 2020). However, information on the effects of pesticides on the growth and respirometric profile of *A. vinelandii* is scarce.

Although there are some studies focused on the role of *Azotobacter* species in tolerance and degradation of pesticides, the information about the effect of OP like CP in the *A. vinelandii* growth and respiratory activity is limited. Therefore, this study aimed to evaluate the growth, respiratory activity, and biodegradation of chlorpyrifos in cultures of *Azotobacter vinelandii* ATCC 12837. A strategy based on the modification of culture media and aeration conditions was carried out to increase the cell concentration of *A. vinelandii*, in order to favor and determine its tolerance to chlorpyrifos and its degradation ability.

## Materials and methods

### Microorganism

Experiments were carried out using *A. vinelandii* ATCC 12837. Cells were cryopreserved at − 70 °C in 40% (w/w) glycerol solution and maintained by monthly subculture on Burk´s-sucrose (BS) agar slopes and stored at 4 °C (Peña et al. 2011).

### Preparation of inoculum

The inoculum was prepared as follows: *A. vinelandii* cells were grown at 29 °C in 250 mL Erlenmeyer flasks, containing 50 mL of BS medium for 24 h at 200 rpm. Flasks were incubated until they reached a biomass concentration of 1 g L^−1^ (measured by dry weight). The liquid culture was diluted at 10% with a fresh BS liquid medium. This suspension was used as inoculum. Each flask was inoculated with 0.1 g L^−1^ of biomass.

### Culture media

Four different media were used for *A. vinelandii* culture with the following composition (g L^−1^): (1) BS: sucrose 20, yeast extract (Difco™ BS, USA) 3, K_2_HPO_4_ 0.66, KH_2_PO_4_ 0.16, NaCl 0.2, MgSO_4_·7H_2_O 0.2, CaSO_4_ 0.05, Na_2_MoO_4_·2H_2_O 0.0029, FeSO_4_·7H_2_O 0.027, MOPS (Sigma Aldrich, USA) [50 mmol]. (2) BS2: the same BS composition except by sucrose (2 g L^−1^). (3) NBRC: Mannitol 5, yeast extract (Difco™ BS, USA) 3, K_2_HPO_4_ 0.7, KH_2_PO_4_ 0.1, MgSO4·7H_2_O 1, MOPS (Sigma Aldrich, USA) [50 mmol]. (4) NBRCm: the same composition except by mannitol (21.3 g L^−1^). The initial pH was adjusted to 7.2 using NaOH 2N solution. To avoid precipitation during autoclaving, the FeSO_4_·7H2O and Na_2_MoO_4_·2H_2_O solutions were separated from the other medium components during sterilization (121 °C, 35 min). The C:N ratio (g mol/g mol) of the BS, BS2; NBRCm, and NBRC media were 29, 5.9, 29, and 21, respectively.

### Culture conditions

Cultures were carried out in 250 mL Erlenmeyer flasks at 200 rpm and maintained at 29 °C for 72 h in an orbital incubator with a shaking diameter of 2.5 cm. In addition to the flasks used for online measurements of respiration activity, cultures were developed in some parallel flasks, three of which were regularly withdrawn (every 6, 12, or 24 h) and submitted to off-line analytical measurements. Cells of *A. vinelandii* were grown in 250 mL Erlenmeyer flasks containing 50 mL of BS, NBRC, and NBRCm media and the culture conditions previously described. The effect of different aeration conditions was evaluated by growing the cells of *A. vinelandii* in 250 mL Erlenmeyer flasks at different filling volumes, containing 10, 20, and 50 mL of BS medium and cultivated as previously described. In order to evaluate the CP effect, cultures were carried out in 250 mL Erlenmeyer flasks containing 50 mL of BS and BS2 culture media with 0, and 500 ppm of technical grade CP (Clorver® 480 EC Versa Agrochemicals, Mexico) and cultivated under the conditions previously described. Uninoculated media with the same concentration of CP were used as a control.

### Measurements of respiration activity

Oxygen transfer rate (OTR) and respiratory quotient (RQ) were determined by a respiration activity monitoring system (RAMOS) (Anderlei and Büchs 2001). During the measuring phase, this device measures the decrease of oxygen partial pressure in the gas phase of closed 250 mL flasks with a sensor mounted in the neck of each flask. From the slope of the oxygen partial pressure curve, the system calculates the OTR (Gomez-Pazarín et al. 2015). RQ was estimated from the quotient between the molar ratio of cumulative CO_2_ production to cumulative O_2_ utilization (Anderlei et al. 2004). The specific oxygen uptake rate (qO_2_) was obtained from the quotient between the OTR_max_ value and the total protein content as previously described by Díaz-Barrera et al. (2011, 2021).

### Analytical determinations

Biomass and alginate concentrations were determined gravimetrically (Peña et al. 1997). The number of colonies forming units mL^−1^ (CFUs) was estimated by plate count (Strobel et al. 2018). Sucrose was assayed for reducing power with 3,5 dinitrosalicylic acid (DNS reagent) (Sigma Aldrich, USA) (Miller 1959). Samples were previously hydrolyzed using β-fructofuranosidase as described by Peña et al. (2011). The protein concentration was determined by the Lowry method using bovine serum albumin as standard (Lowry et al. 1951).

All experiments were carried out by triplicate, and the results presented are the averages of independent samples. When needed, figures and tables show the mean values and standard deviations among replicates. Statistical analysis was carried out using an ANOVA with a multiple comparison Tuckey test (alpha < 0.05).

### Determination of chlorpyrifos (CP) and 3,5,6-trichloro pyridine-2-phenol (TCP)

The extracts resulting from the CP experiments described above (subsection 2.4 Culture conditions) were evaluated to identify and quantify CP and its main metabolites (3,5,6-trichloro pyridine-2-phenol (TCP), O, O-diethyl thiophosphate (DETP), and chlorpyrifos oxon). The samples were filtered through a 25 mm and a 0.22-μm polyvinylidene fluoride (PVDF) membrane and then were diluted 100 and 1000 times with mobile phase prior to CP and its metabolites detection.

Each standard (chlorpyrifos 99.5% N-11459 (Chem Service, USA); 3,5,6-trichloro-2-piridinol (TCP) 99.5% (33972-BCBZ8746) (Sigma Aldrich, USA); DETP (Sigma Aldrich, USA); chlorpyrifos oxon (Sigma Aldrich, USA)) and samples were automatically injected through a Sample-Manager system–FTN Acquity® to equipment of Ultra Performance Liquid Chromatography (UPLC) Acquity® Serie H (Waters Corporation, USA) equipped with a column Acquity® UPLC BEH C18 1.7 µm, 2.1 × 50 mm, in a volume of 5.0 µL (Waters Corporation, USA). The column temperature was kept at 40 °C. The chromatographic conditions were as follows: The mobile phase A was ammonium formate 5 mM, pH 3.0, and mobile phase B was methanol + ammonium formate 5 mM + 0.1% of formic acid at a constant flow rate of 0.35 mL min^−1^, with the following gradient: starting with 83% of solvent A and 17% of solvent B, reaching the 90% of solvent B at 5.5 min and remaining there for 2 min and returning to its first constitution at 7.51 min and remaining there for 2.5 min. With a total running time of 10 min. The autosampler injection needle was rinsed with a mobile phase after each injection. Nitrogen was used as the desolvation gas at a flow rate of 1000 L h^−1^. The desolvation temperature was 600 °C and the source temperature was 150 °C. Argon was used as the collision gas at a flow rate of 0.14 mL min^−1^.

The identification and quantification were performed by means of ESI^+^ (CP) and ESI^−^ (TCP) mode in a Mass Spectrometer Xevo TQ-S and workstation with MassLynx™ 4.1 software (Waters Corporation, USA). Ions were monitored using Multiple Reaction Monitoring (MRM) (Additional file 1: Table S1).

### Mathematical analysis

The specific growth rate (*μ*) was calculated considering the growth from 0 to 12 h of cultivation, the period at which the culture was growing exponentially. The equation used was: *dX/dt* = *μX* where *μ* is the specific growth rate (h^−1^) and X is the cell concentration (g L^−1^) (Klimek and Ollis 1980). The percentage of degradation and the degradation time in which the pesticide concentration was reduced by 50% was calculated (Abraham and Silambarasan 2016) and reported as DT_50_ values. The CP concentration profiles in each of the experiments were fitted to a pseudo-first-order degradation equation Ct = C0*e-kt where Ct is the concentration of the component at time t, C0 is the initial concentration, k is the degradation constant, and t is the time.

## Results

### Growth and respiratory activity of A. vinelandii under different culture media

**Fig. 1:**
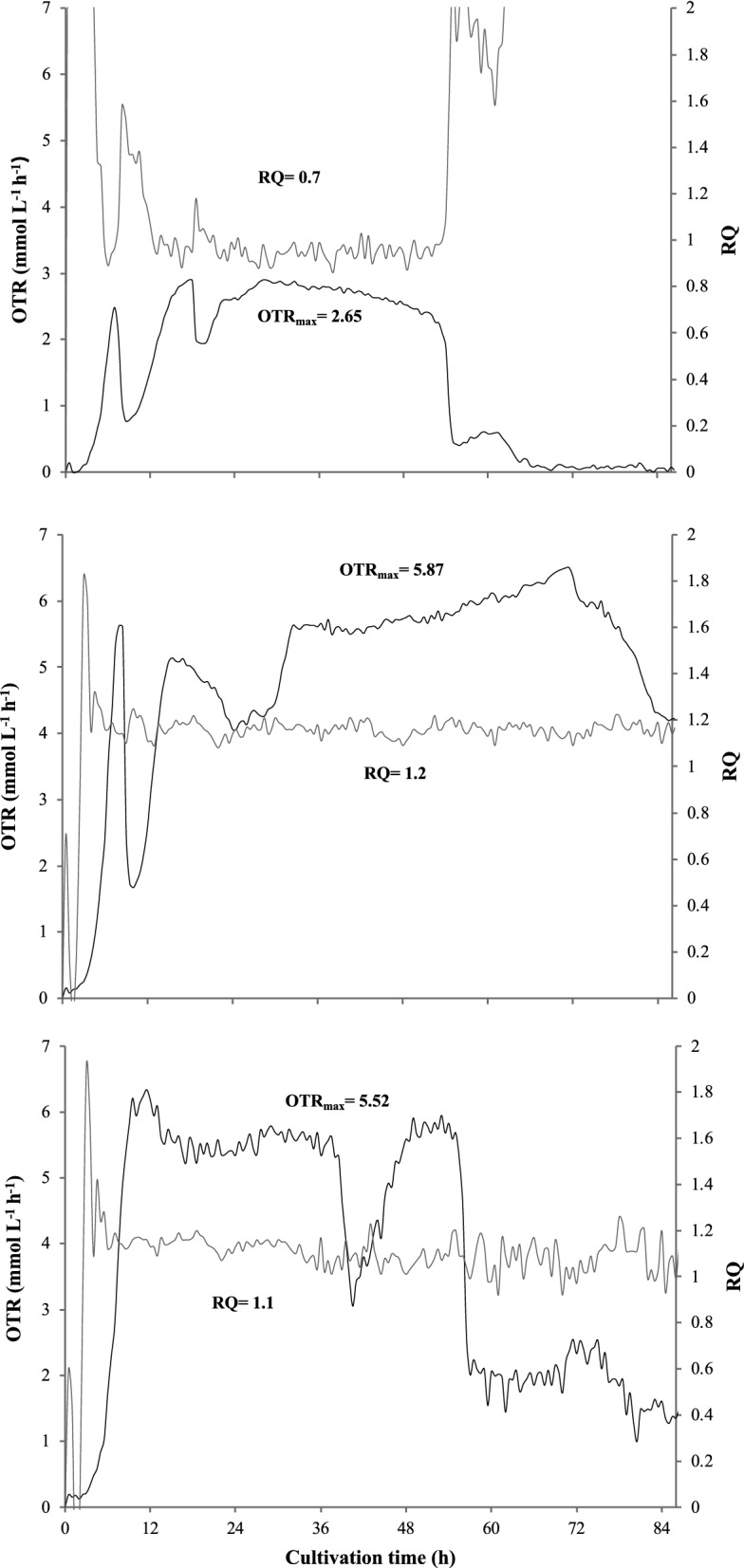
Evolution of the oxygen transfer rate (OTR) and respiration quotient (RQ) in cultures of *A. vinelandii* grown in NBRC (**a**), NBRCm (**b**), and BS (**c**) media

The respiratory activity parameters of *A. vinelandii* developed in NBRC, NBRCm, and BS media are shown in Fig. 1. Both the OTR and RQ profiles were different depending on the amount and type of carbon source present in the different media evaluated. In the three evaluated media, there were notable differences during cultivation time and presented characteristic profiles of oxygen limiting conditions distinguished by a higher sustained OTR value during the cell growth period (OTR_max_). For the NBRC medium with mannitol as carbon source and a C:N ratio 21 (Fig. 1a), variations in OTR were observed at the beginning of cultivation until reaching an average OTR_max_ of 2.65 mmol L^−1^ h^−1^ between 24 and 55 h of culture. With the NBRCm medium (Fig. 1b), increasing the mannitol content (ratio C:N 29), the OTR_max_ increased at 5.87 mmol L^−1^ h^−1^ as expected because of the increase of the carbon source, indicating a high respiration activity and extending the culture time up to 80 h. Finally, in the BS medium (with sucrose as carbon source and a C:N ratio of 29) (Fig. 1c), an exponential increase in OTR was observed from 0 to 12 h, reaching an average OTR_max_ of 5.52 mmol L^−1^ h^−1^ until 36 h, when it decreased and then increased again until 48 h. Concerning the RQ values, in the NBRCm and BS media, similar values were obtained, both above 1 (1.2 and 1.1, respectively) indicating a less oxidative metabolic activity; whereas, the medium with a lower carbon concentration (NBRC) reached an average RQ of 0.7.

**Fig. 2:**
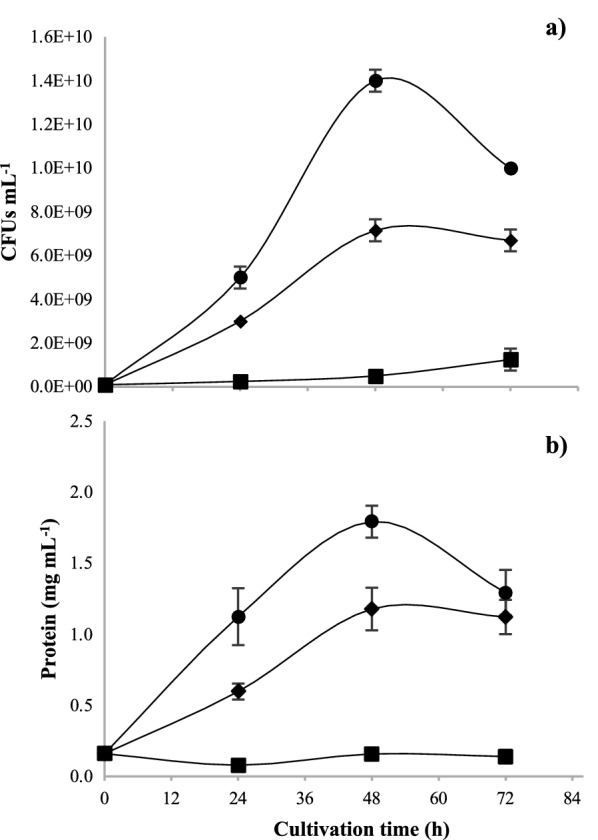
Growth of *A. vinelandii* in different culture media. CFUs (**a**) and protein content (**b**) of *A. vinelandii* cultures in shake flasks in BS (black circle), NBRCm (black diamond suit), and NBRC (Black square) medium. Data are presented as the mean and standard deviation from three experiments

The growth of *A. vinelandii* determined by CFUs and total protein in the different culture media is shown in Fig. 2. The maximum values of CFUs mL^−1^ were 1.4 × 10^10^ at 48 h in the BS medium, followed by NBRCm medium (9.16 × 10^9^) and NBRC (1.25 × 10^9^) at 72 h (Fig. 2a). Similarly, total protein content (Fig. 2 b) increased exponentially up to 48 h and it was higher in BS medium compared to NBRCm medium containing the same C:N ratio (29); whereas cultures with NBRC medium (C:N ratio 21) showed notably lower growth and protein content.

Finally, the kinetic and respirometric parameters of *A. vinelandii* culture on the different media are summarized in Table 1. It is clear from the values of the table that a higher *μ*, number of CFUs, and protein content were reached in the cultures with the BS medium when OTR_max_ was 5.52 mmol L^−1^ h^−1^. It is important to point out that the highest alginate content was obtained in the NBRCm medium, indicating that with mannitol as a carbon source, compared to sucrose medium (BS), the alginate synthesis was improved (5.17 g L^−1^); whereas in BS medium, the alginate production was lower (0.97 g L^−1^alginate).

**Table 1:** Kinetic and respirometric parameters of *A. vinelandii* cultured in shake flasks in different culture media and conditions

Culture medium	Specific growth rate (μ)	Duplication time (h)	Final alginate (gL^−1^)	Protein (mg mL^−1^)	CFUs (mL^−1^)	OTR_max_ (mmol L ^−1^ h^−1^)	qO_2 max_ (mmol /mg mL^−1^ h^−1^)
NBRC	0.03	9.45	0.49 ± 0.03	0.14 ± 0.01	1.25 × 10^9^	2.65	18.92
NBRCm	0.08	6.30	5.17 ± 0.56	1.12 ± 0.06	9.16 × 10^9^	5.87	5.24
BS	0.12	5.55	0.97 ± 0.12	1.79 ± 0.02	1.4 × 10^10^	5.52	3.08

BS medium with different filling volume (mL)	Specific growth rate (μ)	Duplication time (h)	Biomass (g L^−1^)	Protein (mg mL^−1^)	CFUs mL^−1^	OTR_max_ (mmol L^−1^ h^−1^)	qO2_max_ (mmol /mg mL^−1^ h^−1^)
50	0.12	5.77	9.77 ± 0.65	2.78 ± 0.26	1 × 10^10^	5.5	1.97
20	0.08	6.30	4.67 ± 0.18	1.04 ± 0.28	8.2 × 10^8^	11.45	11
10	0.11	5.80	5.90 ± 0.95	2.12 ± 0.05	9.4 × 10^9^	22	10.37

Culture medium	Specific growth rate (μ)	Duplication time (h)	Biomass (g L^−1^)	Protein (mg mL^−1^)	CFUs mL^−1^	OTR_max_ (mmol L^−1^ h^−1^)	qO_2 max_ (mmol /mg mL^−1^ h^−1^)
BS	0.26	2.77	8.9 ± 0.1	1.82 ± 0.21	9 × 10^9^	5.88	2.66
BS + CP	0.26	2.83	10.4 ± 0.05	3.32 ± 0.18	1 × 10^10^	7.9	2.37
BS2	0.14	5.05	2.27 ± 0.2	0.59 ± 0.06	2.1 × 10^7^	5.38	9.11
BS2 + CP	0.19	4.45	2.93 ± 0.02	0.63 ± 0.04	2.51 × 10^7^	6.9	10.9

Experiments were carried out in triplicate and the results presented are the averages and standard deviation of independent runs

### Growth and respiratory activity of A. vinelandii under oxygen and non-oxygen-limited conditions

**Fig. 3:**
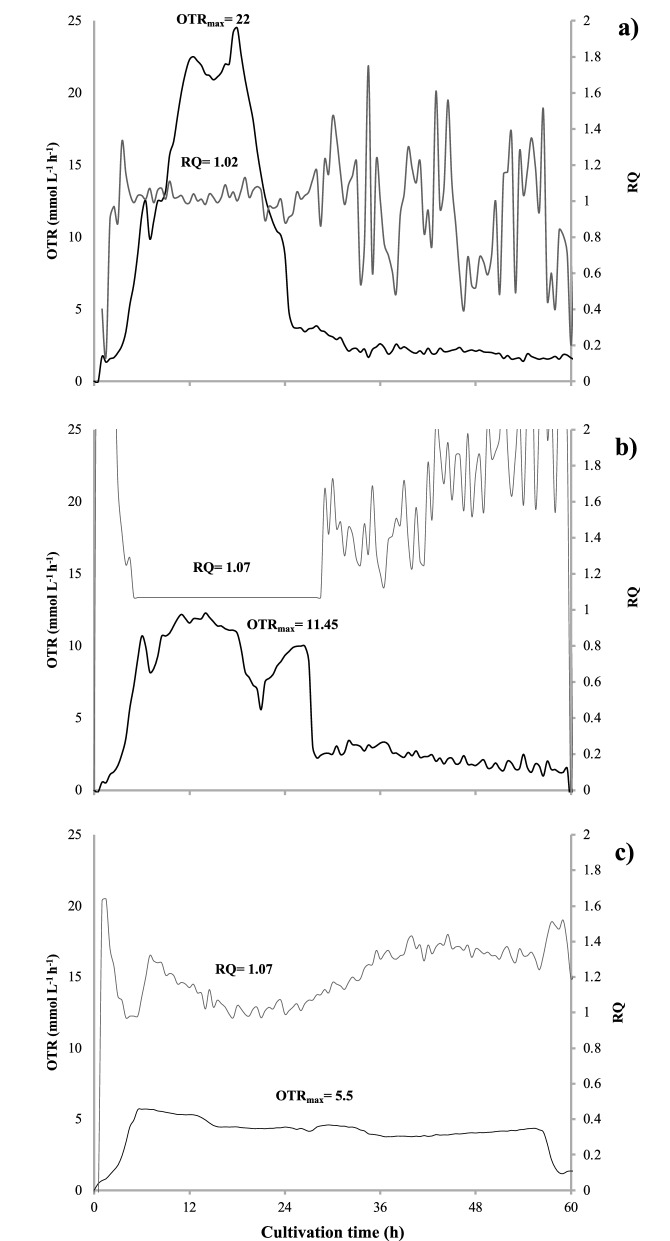
Evolution of the oxygen transfer rate (OTR) and respiration quotient (RQ) in cultures of *A. vinelandii* grown in BS liquid medium with 10 (**a**), 20 (**b**), and 50 mL (**c**) of filling volumes

Figure 3 shows the respiratory activity parameters of *A. vinelandii* developed in BS medium with different filling volumes 10 (a), 20 (b), and 50 mL (c). As it was expected, the OTR_max_ values increased considerably, decreasing the filling volume, obtaining values of 5.5, 11.45, and 22 mmol L^−1^ h^−1^ with 50, 20, and 10 mL, respectively. With 50 and 20 mL, a typical oxygen limitation OTR profile was obtained for *A. vinelandii* cells.

On the other hand, in the cultures with 10 mL of filling volume, a typical non-oxygen-limited profile was observed. In that case, the OTR_max_ was reached at 20 h of culture, followed by a drop in the respiration rate, indicating the decrease in oxidative activity due to the rapid depletion of the sucrose. In the case of the cultures using 20 mL of filling volume, the same drop was presented at 20 h but increased again from 20 to 27 h. Finally, with 50 mL of filling volume, a previously described oxygen limitation profile was exhibited, showing an exponential increase in OTR during the first 6 h of culture and a stationary stage that remained until the carbon source in the medium was exhausted, prolonging the culture until 55 h. In contrast, the RQ was not modified by the filling volume, being in all cases RQ of 1.02–1.07.

**Fig. 4:**
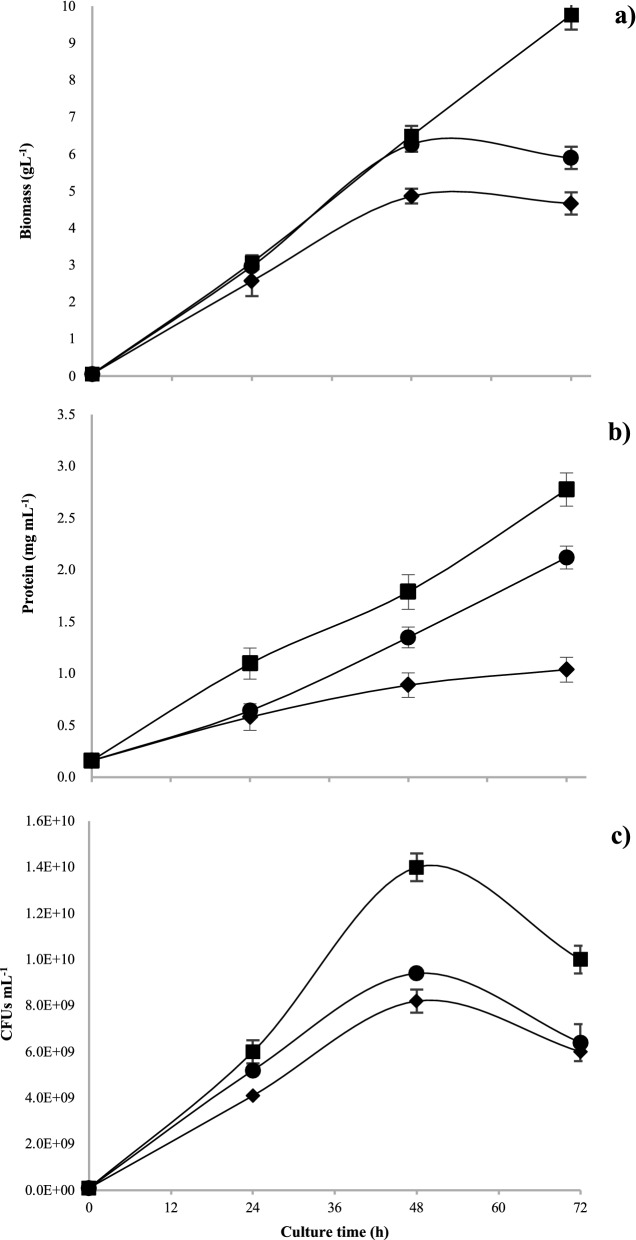
Biomass (**a**), protein content (**b**), and CFUs growth kinetics (**c**) of *A. vinelandii* cultures in 250 mL shake flasks at different filling volumes 10 mL (Black circle), 20 mL (black diamond suit) and 50 mL (black square). Data are presented as the mean and standard deviation from three experiments

As it is presented in Fig. 4, the maximum growth values determined by the parameters of maximum biomass (9.7 g L^−1^), CFUs mL^−1^ (1 × 10^10^), and total protein (2.78 mg mL^−1^) were obtained in the lowest OTR condition, i.e., in the BS medium with 50 mL filling volume between 48 and 72 h of culture. Similarly, the kinetic parameters (Table 1) with that condition showed a higher growth rate of 0.12 h^−1^ and thus a shorter doubling time (5.7 h) even under O_2_-limiting conditions (5.5 mmol L^−1^ h^−1^). It is clear that, the growth of strain ATCC 12837 in BS medium under limited oxygenation conditions did not significantly affect growth, and also presented better cell viability, protein content, and lower qO_2_.

### Growth and respiratory activity of *A. vinelandii* in media with chlorpyrifos

**Fig. 5:**
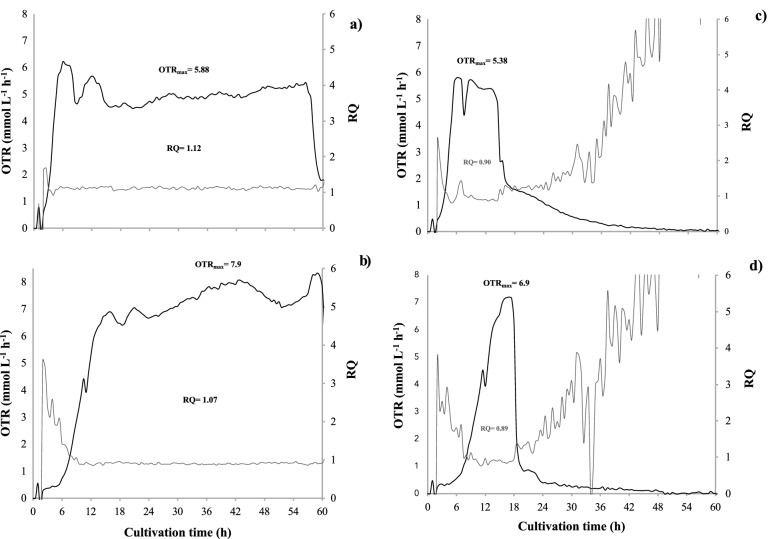
Evolution of the oxygen transfer rate (OTR) and respiration quotient (RQ) in cultures of *A. vinelandii* grown in media: BS (**a**), BS + 500 ppm CP (**b**), BS2 (**c**), and BS2 + 500 ppm CP (**d**)

The respiratory activity parameters of *A. vinelandii* developed in BS and BS2 media with and without CP are shown in Fig. 5. In the first case, when the bacterium was cultured without a decrease in the carbon source (sucrose) in both BS medium without the contaminant (a) and BS with CP (b), clear differences in OTR at the first 12 h of culture were observed. Particularly in the BS medium with CP (Fig. 5b), the increase in the OTR until reaching the maximal was slower compared to the medium without pesticide (BS) (Fig. 5a) in which during the first 6 h the maximum OTR was reached. However, the OTR_max_ values were higher in the medium with CP (7.9 mmol L^−1^ h^−1^) in contrast to the BS medium (5.88 mmol L^−1^ h^−1^), and a prolongation of the respiratory activity up to more than 60 h of culture was observed, suggesting a higher metabolic activity. The average RQ values for the medium with CP were slightly lower (1.07) compared to the BS medium (1.12), in both cases greater than 1. In the second case, by decreasing the concentration of the carbon source (BS2) tenfold and with the addition of the pesticide (B2 + CP) (Fig. 5c and d) a similar behavior to the previous one was obtained in terms of the increase in OTR_max_ for the medium with pesticide (6.9 mmol L^−1^ h^−1^) compared to the BS2 medium (5.38 mmol L^−1^ h^−1^) and a delayed activity in the increase of OTR in the first hours of culture, possibly linked to the adaptation of the bacteria to the presence of the contaminant. Finally, the RQ values for BS2 and BS2 + CP media were 0.90 and 0.89, respectively.

**Fig. 6:**
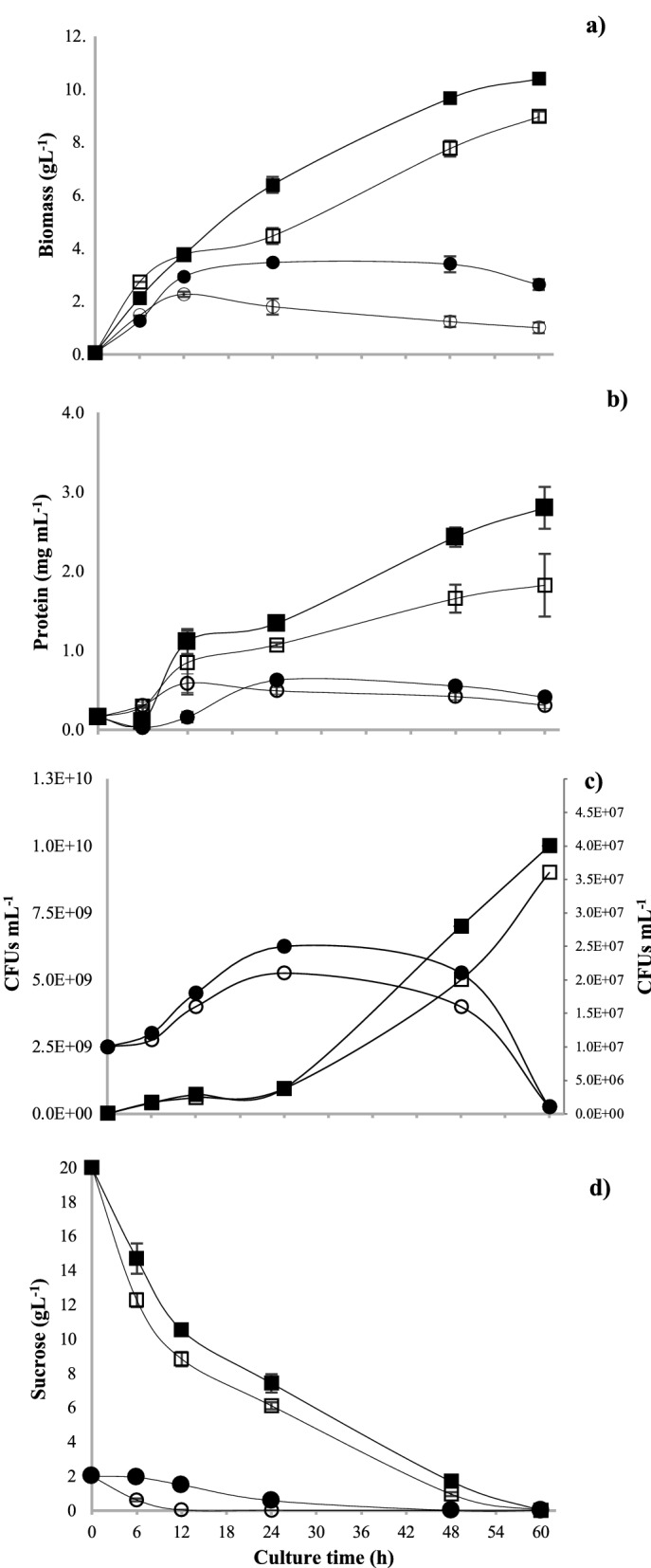
Biomass (**a**), protein content (**b**), CFUs (**c**), and Sucrose consumption (**d**) of *A. vinelandii* growth in BS (white square), BS + CP (black square), BS2 (white square) and BS2 + CP (black circle). Data are presented as the mean and standard deviation from three experiments

Regarding the growth of *A. vinelandii* in BS medium with and without CP, Fig. 6 shows the biomass (a), protein content (b), and CFUs (c), as well as sucrose consumption of all treatments. The BS + CP and BS2 + CP media obtained higher biomass production in relation to the controls without pesticide. The media with 20 gL^−1^ sucrose showed exponential growth until 60 h of culture, while with 2 gL^−1^ sucrose, the exponential phase ended at 12 h of culture. Similarly, the higher total protein content and CFUs mL^−1^ were recorded in the media with CP at 60 and 24 h for BS + CP and BS2 + CP media, respectively.

On the other hand, sucrose consumption (d) was slightly faster in the media without pesticide compared to the media with CP, indicating the use of alternative sources present in the medium with pesticide. Finally, the kinetic parameters showed a growth rate without statistical differences for the media with pesticide and their respective controls, as well as a lower qO_2_ in the BS + CP medium (Table 1).

### Tolerance and biodegradation of chlorpyrifos

The tolerance and biodegradation of CP and its major metabolite TCP were assessed using *A. vinelandii* ATCC 12837 in liquid culture. Although the ATCC 12837 strain was exposed to a concentration of 500 ppm of CP, this demonstrated not only tolerance to the compound but also increased growth and respiratory activity as described above in both BS and BS2 media.

**Fig. 7:**
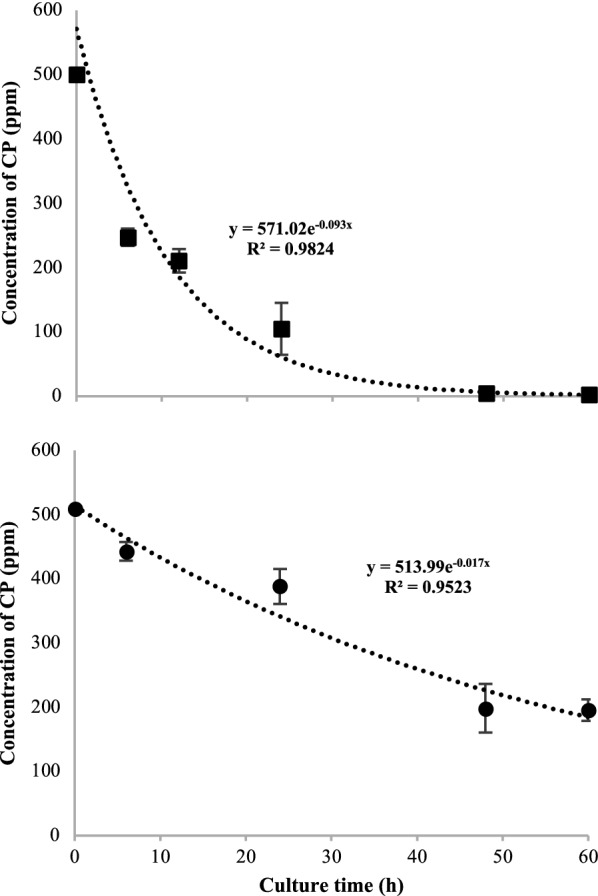
Chlorpyrifos degradation by *A. vinelandii* growth in BS + CP (black square) and BS2 + CP (black circle)

According to the analysis of detection and quantification of CP and its intermediates in the supernatants of *A. vinelandii* cultures, both in BS and BS2 medium, a decrease in the concentration of the pesticide was determined in the medium (Fig. 7). Strain ATCC 12837 completely degraded the pesticide (500 mg L^−1^ of CP) with a DT_50_ of 6 h with BS medium. Whereas in the BS2 medium, when sucrose was reduced, the strain degraded 330 mg L^−1^ of CP and the time to reach DT_50_ was 30 h. The above was influenced by reduced growth in the BS2 medium with low sucrose content. The degradation percentages of the strain after 60 h of culture were 99.5% and 66.8% in BS and BS2 media, respectively.

According to our screening analysis of CP and its main metabolites by UPLC/MS–MS, no accumulation of TCP or formation of other intermediate compounds (DETP or chlorpyrifos oxon) was detected in *A. vinelandii* growth supernatants with 500 ppm CP. Under the conditions tested, the bacteria apparently can metabolize CP and use it for growth and energy. In addition, a higher percentage of degradation was observed when grown on a nutrient-rich medium (BS medium).

## Discussions

### Growth and respiratory activity of *A. vinelandii* in different culture media

Regarding to the respiratory activity *A. vinelandii* grown under different culture media, the OTR and RQ were dependent on the amount and type of carbon source available (Fig. 1), a similar behavior that has been observed previously (Noguez et al. 2008). This is explained because both parameters are substrate-dependent (Kahraman and Altin et al. 2020). Factors such as oxygen availability and the amount or type of carbon source modify the metabolic response of aerobic organisms to oxidize compounds and produce CO_2_ which can be monitored by OTR and RQ values (Gomez-Pazarín et al. 2015).

In this context, characteristic profiles of oxygen limiting conditions were observed in the three evaluated media, similar to those previously reported for cultures of *A. vinelandii* (Peña et al. 2011)*.* Those profiles are characterized by maintaining a sustained OTR_max_ value during the culture time and RQ values of 1 or higher, as those obtained in the NBRCm and BS media. RQ values higher than 1 are generally attributed to anaerobic or microaerophilic conditions, where the oxygen availability is not sufficient to oxidize the carbon source present in the media, whereas, values below 1 are related to aerobic processes (Dilly 2001, 2003; Lamy et al. 2013).

In our results, the OTR_max_ values and the growth of *A. vinelandii* determined by CFUs and total protein in the different culture media were higher when C:N ratio of 29 was used (Fig. 2). It is known that high concentrations of organic carbon in the form of sugars, alcohols, and organic acids (25%) are used to improve the growth of *Azotobacter* (Tejera et al. 2005). In contrast, Castillo et al. (2017) found that using ratios between 16 and 32 gC gN^−1^, there were no significant differences in the growth of *A. vinelandii* when using sucrose and yeast extract as carbon and nitrogen sources, respectively. In our results with *A. vinelandii* ATCC 12837, the type of carbon source and the increase of the C:N ratio, positively impacted the cell growth, viability, and respirometric parameters, even in oxygen limiting conditions.

In this line, recently Díaz-Barrera et al. (2021) reported that, under oxygen limitation conditions (5.0 ± 0.9 mmol L^−1^ h^−1^) and no-nitrogen fixation, similar to those carried out in our study, *A. vinelandii* ATCC 9046 channeled the carbon source mainly to the production of biomass and intracellular polymers like PHB.

The above is consistent with that reported by Peña et al. (2007, 2011), who obtained higher viability, biomass production, and specific growth rate with the increase in the OTR in cultures in shaken flasks (OTR_max_ 6 mmol L^−1^ h^−1^ compared to an OTR_max_ of 2.5 mmol L^−1^ h^−1^). Also, when *A. vinelandii* was grown at OTR_max_ of 5.5 mmol L^−1^ h^−1^, (similar to that obtained in our study for BS and NBRCm medium) the carbon source was mainly directed to growth with an increase in the biomass concentration, polymer, and CO_2_ production, which may be affected by the strain qO_2_ (Diaz-Barrera et al. 2011). That is coherent with our results because a lower qO_2_ was obtained in the BS medium, the same with the higher growth and respiratory activity.

### Growth and respiratory activity of *A. vinelandii* under oxygen and non-oxygen-limited conditions

The OTR_max_ values in the cultures of *A. vinelandii* in BS medium increased considerably by decreasing the filling volume (Fig. 3), obtaining a typical oxygen limitation and non-oxygen limitation profiles as previously reported in cultures of *A. vinelandii* in stirrer tank and shaken flasks (Peña et al. 2007; Díaz-Barrera et al. 2007; Moral et al. 2016). In contrast, the RQ values were not modified by the filling volume. These data contrast with those previously reported for *A. vinelandii* ATCC 9046 (Peña et al. 2011), where at higher OTR, RQ values are less than 1 and conversely at low OTR, higher than 1. This could be related to a lower respiration rate observed in the strain ATCC 12837 used in the present study. For the case of *A. vinelandii* ATCC 9046 strain, it has been documented that it possesses mechanisms that regulate its respiration efficiency depending on the modifications of its respiratory chain by the activation or deactivation of terminal oxidases. These oxidases respond to environmental and nutritional changes such as oxygen availability to maximize energy conservation or produce intracellular or extracellular polymers and it may vary slightly among strains (Castillo et al. 2020).

On the other hand, previous studies with *A. vinelandii* in shaken flasks, under high and low aeration conditions, showed that changes in oxygen availability had a considerable impact on the growth profiles (Castillo et al. 2013), especially on growth, measured as biomass, and protein production (Peña et al. 2011). In the study of García et al. (2018), the highest protein yield of *A. vinelandii* (0.15 g protein g glucose^−1^) was obtained in the cultures developed under the lowest OTR (2.4 mmol L^−1^ h^−1^). In our case, the maximum growth values were also obtained at the lowest OTR condition (Fig. 4). Other authors have reported that when OTR_max_ was reduced, the *μ* value also decreased, although without changes in the final biomass concentration (Peña et al. 2007, 2011; Díaz-Barrera et al. 2021). In our study, the growth of strain ATCC 12837 in BS medium under limited oxygenation conditions did not significantly affect growth, and also presented better cell viability, protein content, and lower qO_2_. The last is a relevant characteristic to consider as a scale-up criterion, owed to the high requirements that characterize other strains like *A. vinelandii* ATCC 9046, and are usually a limiting condition for process scale-up.

### Growth and respiratory activity of *A. vinelandii* in media with chlorpyrifos

Due to the typically high respiration rates of *A. vinelandii*, oxygen limitations and preferences for carbon sources usually occur in the early stages of fermentation (Peña et al. 2007). Besides, in the presence of toxic substances, a period of adaptation or reduced respiration rates could occur early in the culture (Chennappa et al. 2013). In our case, we observed differences in OTR values in the cultures of *A. vinelandii* developed in BS and BS2 media with CP at the first 12 h of cultivation, in OTR_max_, and the prolongation of respiratory activity, relative to their respective control conditions (Fig. 5).

It is important to point out that, this is the first time when OTR and RQ online values have been estimated for *A. vinelandii* in response to the presence of CP in a liquid medium with sucrose. The OTR_max_ values recorded were significantly higher in media with the pesticide, and prolongation of respiratory activity was observed in both conditions (BS and BS2). The above suggested an increase in metabolic activity related to the addition of the pesticide as a carbon source.

Regarding the RQ values, the average values were slightly lower by decreasing tenfold the carbon concentration (BS2) with and without the addition of pesticide. The latter was related with the decrease of the carbon availability and presence of pesticide, which resulted in an oxygen non-limiting condition, where the lower metabolic activity and CO_2_ production was reflected in the RQ value (Lamy et al. 2013). All the above was also then supported by growth parameters.

In the cultures of *A. vinelandii* in the presence of CP, the growth was better than when the pesticide was not used (Fig. 6). This suggests the use of CP as a carbon source by *A. vinelandii* ATCC 12837, since it not only tolerated the high concentration of the pesticide (500 ppm) but also had a significantly higher growth compared to the reference treatments, as well as a lower qO_2_ in the BS + CP medium. This is consistent with other *Azotobacter* strains that did not show any in vitro growth impairment in the presence of CP (Chennappa et al. 2014a). On the other hand, slightly faster sucrose consumption in the media without pesticide, indicating the use of alternative sources present in the medium with CP.

Although other *Azotobacter* strains have shown tolerance to CP (Gurikar et al. 2016; Chennappa et al. 2019), this is the first time that respirometric parameters are measured and related to the growth of *A. vinelandii* in presence of a pesticide. In the present study, we highlight that strain ATCC 12837 growth in a high CP concentration in contrast with previously reported (100 ppm and 1–5%) (Mac-Rae and Celo 1974; Chennappa et al. 2019); without adversely affecting its growth or respiratory activity. In contrast, according to Mac-Rae and Celo (1974), despite showing tolerance, the oxygen consumption rate of *A. vinelandii* was considerably reduced when using 100 ppm of OP (Naled, Terracur-P, coumaphos, malathion, CP). This could be related to the strain ATCC 12837 high tolerance to the pollutant, since decrease in its respiration activity is not observed as a result of exposure to CP.

### Tolerance and biodegradation of chlorpyrifos

The tolerance and biodegradation of toxic compounds by *Azotobacter* spp. have not been fully addressed, especially concerning pesticides. Recently, it has been suggested that strains of this genus can show tolerance to compounds such as CP, and even degrade it (Chennappa et al. 2019). In the present study, even though the strain was exposed to a concentration of 500 ppm of CP, higher than those used in other reports for *Azotobacter* spp. and other genera (from 10 to 300 ppm) (Maya et al. 2011; Rayu et al. 2017; Akbar and Sultan et al. 2016; Liu et al. 2011: Yang et al. 2005; Abraham and Silambarasan 2016; Shi et al. 2019), the strain ATCC 12837 demonstrated not only tolerance to the compound, but also increased growth and respiration activity.

Commonly, some microorganisms can be tolerant to low concentrations of pesticides such as CP, thanks to primary protective mechanisms mediated by oxidative enzymes as cytochrome p450, peroxidases, and polyphenol oxidases (Abraham and Lambarasan 2018), but high CP concentrations could strongly affect the bacterial growth (Singh et al. 2011) and drastically decrease the number of tolerant organisms at concentrations above 100 ppm (Hernández-Ruíz et al. 2017).

In addition, one of the limiting factors in the complete degradation of CP is usually the generation of secondary metabolites such as TCP. TCP is the main degradation product of CP and tends to be resistant to biodegradation or bactericidal due to its composition, as it contains a pyridinol ring with 3 chlorine atoms (Jabeen et al. 2015). The above limits the number of organisms capable of fully mineralizing the compound (Abraham and Silambarasan 2016). Some strains, such as *Pseudomonas* sp. and *Bacillus megaterium*, have been able to degrade CP (100 mg L^−1^) but not completely TCP (Barman et al. 2014; Zhu et al. 2019). This situation may be reflected with the accumulation of TCP and other intermediates, which prevents the complete elimination of the parent compound (Barman et al. 2014) and may allow further dissipation of contaminants, as e.g. TCP is more soluble than the parent molecule (John and Shaike 2015) and acts as an endocrine disruptor (Fishel 2013).

In our study, *A. vinelandii* tolerated, grew, and efficiently degrade a high CP concentration in vitro, both in BS and BS2 media, without the accumulation of TCP or formation of other metabolites (DETP or chlorpyrifos oxon). This suggests that, under the conditions tested, the bacteria can completely metabolize CP and use it for growth and energy. However, the mechanisms of CP degradation or the involvement of enzymes associated with its degradation such as organophosphate hydrolases (Li et al. 2007; Barman et al. 2014) have not yet been fully elucidated or reported for *Azotobacter* spp.

One of the closest genera to *Azotobacter* that has shown efficiency in CP degradation is *Pseudomonas*, as it can use it as a carbon source and energy (Gilani et al. 2016); and it has been particularly documented in strains such as ATCC 700113 (Feng et al. 1998). *Pseudomonas syringae* was able to degrade 99.1% of 100 mg L^−1^ of CP in 5 d also presenting degradation-associated phosphoesterase enzymatic activity (Zhu et al. 2019). However, the initial concentration is important and another limiting factor in CP degradation. e.g., although *Pseudomonas* spp. can degrade CP, it decreases its growth or stops degrading TCP at concentrations higher than 200 ppm (Li et al. 2007). In contrast, *A. vinelandii* ATTCC 12837 strain showed higher tolerance (500 ppm) and degradation efficiency compared to *Pseudomonas* spp.

On the other hand, *Pseudomonas putida* is among the most efficient strains in CP degradation (Gilani et al. 2016). Especially when it was developed under optimal growth conditions in glucose supplemented medium (Vijayalakshmi and Usha 2012). In the case of *A. vinelandii*, a higher percentage of degradation was observed when grown on a nutrient-rich medium (BS medium) (Fig. 7). This is consistent with what was also reported by Gilani et al. (2010), who point out that the degradation of CP in the presence of nutrients increases due to better cell growth by greater availability of easily metabolizable compounds, which allows the pesticide degradation in a co-substrate condition.

Furthermore, it has been described that, under neutral pH conditions, as our experiment was conducted, CP can be hydrolyzed and follow different biodegradation pathways; and under aerobic conditions, the breaking of the aromatic rings is favored (Jayasri et al. 2014).

**Fig. 8:**
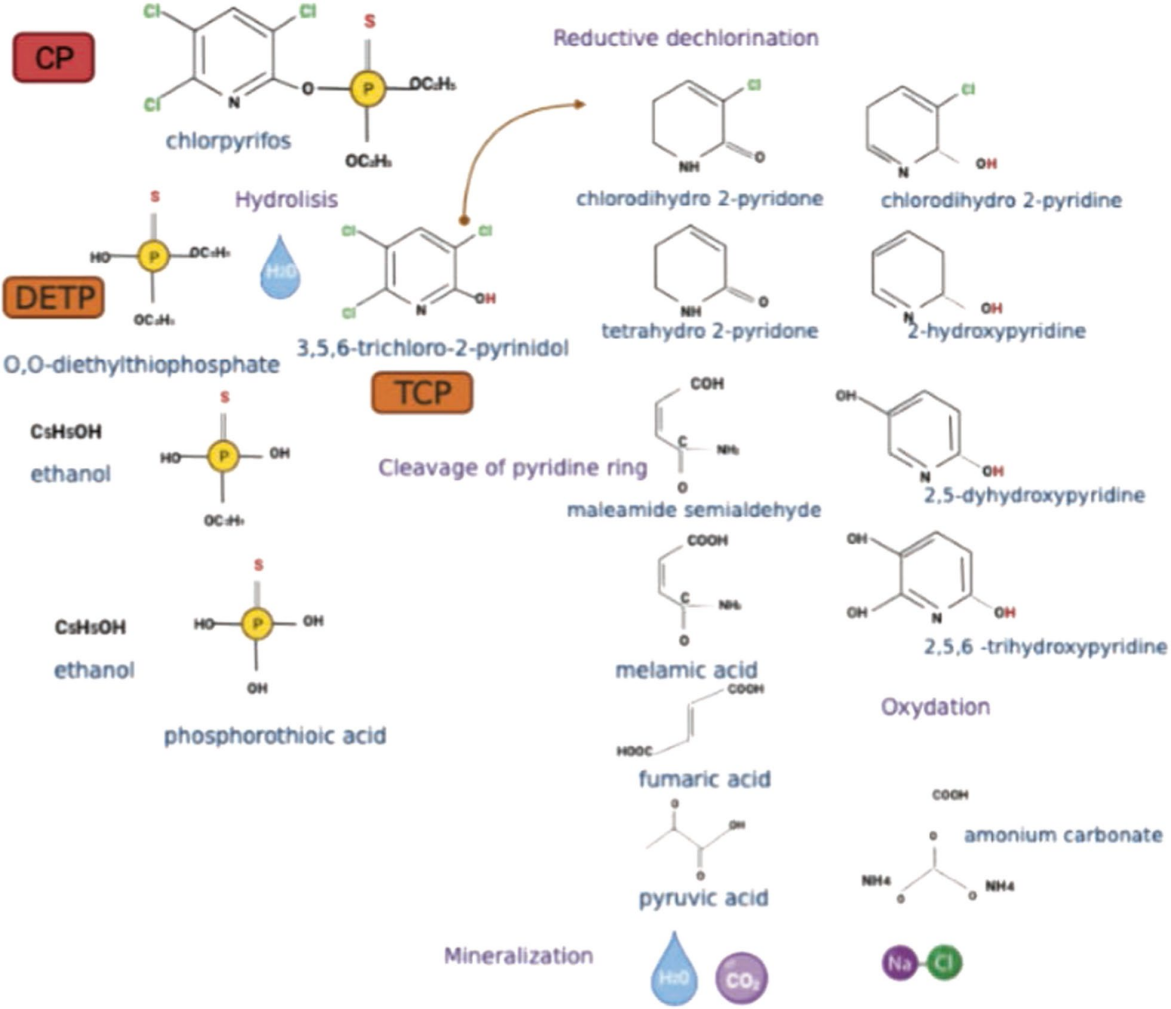
Chlorpyrifos biodegradation pathway (own elaboration)

Given that in our screening analysis of CP and TCP by UPLC/MS–MS no other intermediate compounds were detectable during in vitro culture development, we show a possible degradation pathway that *A. vinelandii* ATCC 12837 could follow (Fig. 8). The hydrolysis of CP to TCP, followed by reductive dechlorination of TCP and incorporation of the pyridine ring into the Krebs cycle which completes the degradation of CP and this has also been identified in *Pseudomonas* (Vijayalakshmi and Usha 2012); or the formation of DETP which is rapidly degraded to ethanol and phosphorothioic acid molecules and can be used as a S, N, and P source for microorganisms (Rokade and Mali 2013; Bose et al. 2021).

Regarding efficiency, in our study, *A vinelandii* degraded CP 10 times faster (200 mg L^−1^ in 4.8 h in BS medium), compared to the bacterium *Cupriavidus nantogensis* (200 mg L^−1^ in 48 h) and similarly could tolerate up to 500 mg L^−1^ (Shi et al. 2019). On the other hand, the fungus *Cladosporium cladosporioides* degraded only 50 ppm of CP in 5 d and tolerated 500 mgL^−1^ as well, and although it generated TCP as an intermediate, it degraded rapidly without leading to accumulation; and similar to our results, they did not detect traces of compounds in chromatographic analysis (Chen et al. 2012).

The elimination of CP in the medium suggests that the strain utilizes the pesticide as a carbon source and energy efficiently compared to other strains in addition to being highly tolerant so it could maintain its activities as a PGPR. This has been previously described in *Azotobacter salinestris* which maintained the highest production of indoleacetic acid (auxin) on medium supplemented with 1 mg tryptophan and CP (1%), indicating that CP did not negatively affect its growth or phytohormone synthesis (Chennappa et al. 2016).

It is worth noting that, decades of research on the effect of pesticides such as CP on the development of *Azotobacter* spp. generally reported growth impairment, respiratory inhibition, changes in oxygen consumption rate_,_ and no degradation (Mac-Rae and Celo 1974; Omar and Abd-Alla 1992). Recent studies have identified that certain strains have shown greater tolerance to different compounds, particularly to CP (Chennappa et al. 2014a; Farhan et al. 2021). This is attributable, according to some authors, to the fact that rhizospheric microorganisms that have been chronically exposed to pesticides have created resistance and accumulated adaptations to use them as a carbon source and energy (Roy et al. 2020); while, maintaining and even favoring their PGPR activities (Shahgholi and Ahangar 2014; Pant et al. 2016).

This allows that *A. vinelandii* ATTCC 12837 to be an excellent candidate to be used in CP remediation, both in vitro and in situ, since microorganisms that can degrade pesticides in vitro usually maintain this capacity in soil (Vidya Lakshmi et al. 2009); although considering a decrease in the speed and efficiency of degradation (Deng et al. 2015) due to multiple edaphoclimatic factors that may vary their behavior (Aasfar et al. 2021).

In conclusion, the excessive use of pesticides as CP is related to multiple environmental alterations. Degradation strategies using rhizospheric microorganisms that also favor the development of crops have become more interesting in the search for alternatives that contribute to the reduction of fertilizers and toxic agents. In this context, we proposed a cultivation strategy to evaluate the growth of *A. vinelandii* ATTC 12837 and the degradation of CP. Our strategy to optimize bacterial growth allowed us to demonstrate that sucrose as a carbon source favored the in vitro development of *A. vinelandii* ATCC 12837, as well as the degradation of CP. Furthermore, despite the high oxygen consumption rates that are often a limiting step for large-scale production of *Azotobacter* spp., oxygen-limiting conditions did not affect the growth of ATTC 12837 strain. Also, this is the first time when online respirometric parameters have been estimated in response to the presence of CP for this bacterium. On the other hand, the results demonstrate that the model organism *A. vinelandii* ATTC 12837 (deeply studied as a PGPR), is also highly tolerant and efficiently degraded chlorpyrifos, without accumulation of toxic secondary metabolites, and with the potential to develop into a promising candidate for improving the productivity of crops in pesticide-contaminated soils.

## Supplementary Information

**Additional file 1: Table S1.** Tandem MS conditions.

## Data Availability

The datasets generated during and/or analyzed during the current study are available from the corresponding author on reasonable request.
