# Supplementary material for: Growth, respiratory activity and chlorpyrifos biodegradation in cultures of Azotobacter vinelandii ATCC 12837
Source: AMB Express. 2021 Dec 27;11:177. doi: 10.1186/s13568-021-01339-w (PMC8712287; doi:10.1186/s13568-021-01339-w)
Supplement: Supplementary file 1 — Additional file 1: Table S1. Tandem MS conditions. [file 13568_2021_1339_MOESM1_ESM.docx]

**Journal: “AMB Express”**

**Manuscript title: “Growth, respiratory activity and chlorpyrifos biodegradation in cultures of *Azotobacter vinelandii* ATCC 12837”**

**Victoria Conde-Avila^1^, Carlos Peña^2^, Beatriz Pérez Armendáriz^1^, Octavio Loera^3^, Carmen Martínez Valenzuela^4^, José Belisario Leyva Morales^4^, Pedro de Jesús Bastidas Bastidas^5^, Holjes Salgado-Lugo^2^, Luis Daniel Ortega-Martínez^1^***

^1^ Facultad de Biotecnología, Universidad Popular Autónoma del Estado de Puebla, México. 13 Poniente No. 1927 Col. Barrio de Santiago, C.P.72410, Puebla, Pue. México.

^2^Departamento de Ingeniería Celular y Biocatálisis, Instituto de Biotecnología, Universidad Nacional Autónoma de México. Apdo. Post. 510-3 Cuernavaca, 62250 Morelos, México.

^3^ Universidad Autónoma Metropolitana-Unidad Iztapalapa. Av. San Rafael Atlixco 186., 09340 Ciudad de México, CDMX.

^4^Doctorado en Sustentabilidad, Universidad Autónoma de Occidente, Unidad Guasave. Avenida Universidad S/N, Fraccionamiento Villa Universidad, C.P. 81048, Guasave, Sinaloa, México.

^5^ Centro de Investigación en Alimentación y Desarrollo, A.C. (Unidad Culiacán). Carretera a El dorado km. 5.5, Campo el Diez, Culiacán, Sinaloa, México, C.P. 80129.

*** Correspondence:**

Luis Daniel Ortega-Martínez

E-mail [luisdaniel.ortega@upaep.mx](mailto:luisdaniel.ortega@upaep.mx)

Phone: +552222299400

**Table S1.** Tandem MS conditions.

| **Analyte** | **Parent m/z** | **Daughter m/z** | **Dwell (s)** | **Cone (V)** | **Collision (V)** |  |
| --- | --- | --- | --- | --- | --- | --- |
| Chlorpyrifos | 349.90 | 97.00  198.00 | 0.115  0.115 | 27  27 | 32  20 | |
| TCP | 196.00  198.00  200.00 | 196.00  198.00  200.00 | 0.200  0.200  0.200 | 30  30  30 | 5  5  5 | |
